# Novel *Mycobacterium tuberculosis* Complex Genotype Related to *M. caprae*

**DOI:** 10.3201/eid2807.212353

**Published:** 2022-07

**Authors:** Joseph Shea, Carol Smith, Tanya A. Halse, Donna Kohlerschmidt, Amy K. Rourke, Kimberlee A. Musser, Vincent Escuyer, Pascal Lapierre

**Affiliations:** Wadsworth Center, New York State Department of Health, Albany, New York, USA

**Keywords:** Mycobacterium tuberculosis, Mycobacterium tuberculosis complex, bacteria, tuberculosis and other mycobacteria, genotypes, animal-adapted, MTBC lineages, United States

## Abstract

We report the unusual genotypic characterization of a bacterium isolated from a clinical sample of a patient who grew up in Bangladesh and lives in the United States. Using whole-genome sequencing, we identified the bacterium as a member of the *Mycobacterium tuberculosis* complex (MTBC). Phylogenetic placement of this strain suggests a new MTBC genotype. Even though it had the same spoligotype as *M. caprae *strains, single-nucleotide polymorphism–based phylogenetic analysis placed the isolate as a sister lineage distinct from *M. caprae*, most closely related to 5 previously sequenced genomes isolated from primates and elephants in Asia. We propose a new animal-associated lineage, La4, within MTBC.

The *Mycobacterium tuberculosis* complex (MTBC) comprises multiple species, divided into human-adapted (*M. tuberculosis* and *M. africanum*) and animal-adapted (*M. bovis*, *M. orygis*, *M. caprae*, and others) tuberculosis (TB) lineages (*1*); L8, one of the most recently described, is most likely human-adapted (*2*). Human-adapted TB has been found to cause disease in certain nonhuman animals and vice versa, but some animal-adapted MTBC species (e.g., *M. surricattae*, dassie bacillus, chimpanzee bacillus) have not yet been reported to cause disease in humans (*3*). Several MTBC species and lineages have been newly reported in recent years, in part because of increased global use of highly discriminatory genotyping methods (*2*,*4*,*5*). Whole-genome sequencing (WGS) has helped classify previously misclassified or undetected rare strains, thus helping to fill gaps in the evolutionary history of TB.

In 2020, the Wadsworth Center at the New York State Department of Health (Albany, New York, USA) received an MTBC isolate from the New York City Public Health Laboratory for routine genotyping and antimicrobial resistance profiling. This isolate was cultured from a sputum sample collected from a 70-year-old patient who grew up in Bangladesh and immigrated to the United States in 2002. The patient was diagnosed with tuberculosis in 2019, 17 years after immigrating to the United States. Unpasteurized milk is a route of infection known for some TB lineages, of note *M. bovis,* and suspected for other MTBC species (*6*,*7*). The patient self-reported a childhood history of drinking raw milk but did not specify the animal source of the milk. PCR screening of the regions of difference (RD) of this isolate revealed a pattern atypical of any known species (*8*).

As part of our diagnostic workflow, we used WGS to identify the bacterium from the sample and determine its antimicrobial resistance profile and genotype, including in silico spoligotype. Our analysis revealed that this isolate was not closely related to any of >4,000 previously sequenced clinical strains in the Wadsworth Center collection. We compared results of phylogenetic analyses of this strain, designated 20-2359 by our laboratory information management system, with phylogenic characteristics from a diverse group of representative strains of *M. caprae*, *M. bovis*, and other *Mycobacterium* spp. gathered from publicly available databases.

## Methods

### PCR-Based Identification

We assessed strain 20-2359 using an in-house developed IS6110-targeted real-time PCR to confirm the identity to the MTBC level and to check for inhibition (*9*). We also ran PCR to differentiate *M. tuberculosis*, *M. bovis*, *M. bovis* bacillus Calmette-Guérin, *M. africanum*, *M. microti*, and *M. canettii*, based on the presence or absence of RD1, RD4, RD9, RD12, and a region exterior to RD9, according to protocols described elsewhere (*8*).

### WGS

We extracted DNA from 1 mL of heat-treated culture material (7H9 broth) using the InstaGene and FastPrep methods described elsewhere (*10*) and prepared sequencing libraries for the Illumina MiSeq platform using Nextera XT (https://www.illumina.com) paired-end 250 bp with 15 PCR cycles for the indexing step, as described elsewhere (*11*). We also performed nanopore sequencing on the Oxford Nanopore MinION platform using the SQK-LSK109 ligation sequencing kit (https://nanoporetech.com), as described elsewhere (*12*).

### Bioinformatics Analyses

We retrieved complete genome sequences of diverse *Mycobacterium* spp. lineages from the National Center for Biotechnology Information (NCBI; https://www.ncbi.nlm.nih.gov) and generated synthetic 250 bp paired-end read sets for pipeline analyses using ArtificialFastqGenerator version 1.0.0 (https://sourceforge.net/projects/old-software-collection/files) (*13*). In addition, for analyses, we downloaded from NCBI Sequence Read Archive (SRA; https://www.ncbi.nlm.nih.gov/sra) reads for animal-associated *M. caprae* and other close MTBC relatives, lineages La2 and La1.1, as described in the recently revised nomenclature (*14*). We analyzed the sequence reads as described elsewhere (*10*) using the Wadsworth Center TB WGS bioinformatics pipeline, which includes a combination read classifications, using Kraken (*15*) and the presence or absence of specific genomic markers to determine the species and lineages of the bacteria from the sample. We screened for the presence or absence of 43 CRISPR spacers in the read sets to determine in silico spoligotyping. We mapped reads to a reference sequence, *M. tuberculosis* H37Rv, to construct consensus sequences, SNP alignments, and phylogenetic reconstructions. After completing mapping, we masked all repeated genomic regions and phage-associated loci to avoid erroneous SNP calling. We generated the SNP matrix using snp-dists (https://github.com/tseemann/snp-dists) and used Unicycler version 0.4.8-β (https://github.com/rrwick/Unicycler) with default parameters, as described elsewhere (*16*), for hybrid de novo assembly and polishing of 20-2359 using the MiSeq and MinION reads. We annotated the 20-2359 genome with pgap build5508 (https://github.com/ncbi/pgap/releases) (*17*) after trimming Illumina adaptors with bbuk from the package BBMap version 38.18 (sourceforge.net/projects/bbmap). We assembled a total of 19 contigs (N50: 476,048 bp) with a length of 4,286,739 bp and 4,015 predicted genes.

We generated phylogenetic trees from the SNP alignments using IQ-TREE version 1.6.12, with automatic best model selection transversion plus empirical base frequencies plus ascertainment bias correction plus FreeRate model with 2 categories base substitution model, and with 1,000 bootstrap support calculations (*18*). RD were bioinformatically determined using RD-Analyzer version 1.01 (*19*). The tree was rooted using the branch leading to the *M. tuberculosis, M. africanum*, *M. microti*, and *M. orygis* clusters.

### Sequencing Reads, Genome Assembly, and Culture Availability

The raw sequencing reads and final genome assembly of strain 20-2359 are available at NCBI under Bioproject PRJNA771604 and nucleotide assembly JAJEJL000000000. Culture of strain 20-2359 will be available from our collection on request to the corresponding author.

## Results

Initial PCR screening of 20-2359 for RD pattern yielded atypical results. Of note, RD1 was present but RD9 did not show any amplification. RD4 and RD12 had late amplification, suggesting possible mutations in the primer or probe sites of this assay, or insertions and deletions impacting the amplicon size of the targets. WGS analysis returned atypical results for identification as well. Species identification with Kraken using a local *Mycobacterium* spp. database, reported 20-2359 as *M. bovis,* although with a low percentage of specific reads. In silico–derived spoligotype listed this strain in the most up-to-date databases as most likely *M. caprae*. This rare spoligotype, 0000000000000000111111111110111111111100000, had previously been reported as *M. bovis* or *M. bovis* subspecies *caprae*–type before *M. caprae* was reported as a unique species. Three other samples in our dataset isolated from primates in China (NCBI SRA nos. SRR1792164, SRR1792165, and SRR7617662) also shared this spoligotype with 20-2359 (Appendix Table 1).

An in-house lineage identification scheme using specific SNPs also failed to positively identify the isolate (Table). We found that 20-2359 lacked 1 of 2 specific mutations required to be classified as either *M. bovis* or *M. caprae* and detected none of the known lineage-specific markers. The same markers were also missing from the monkey and elephant isolates. Genomic analyses of RD confirmed that RD1 was present, but RD4 and RD9 regions were deleted in 20-2359. A more comprehensive analysis of RD in 20-2359 using RD-Analyzer (https://rdanalyzer.com) revealed a presence-absence RD pattern identical to other *M. bovis*–related strains and 1 *M. caprae* strain, NCBI SRA no. ERR1462578 (Appendix Table 2). A closer look at the RD4 sequences for the specific region Rv1496–Rv1518 in *M. tuberculosis* H37Rv in 20-2359 revealed a genomic deletion in 20-2359 different from all other sequences in our dataset, resulting in a unique gene cluster when compared with the other lineages (results not shown). RD4 was deleted in 20-2359, but present in the 5 closely related strains belonging to the proposed La4 lineage, which had complete RD4 and RD patterns identical to *M. caprae*.

**Table:** List of markers used for species and lineage determination in investigation of novel *Mycobacterium tuberculosis* complex genotype related to M. caprae*

Species and lineage	Specific markers	Strain 20-2359 genotype
*Mycobacterium tuberculosis*	gyrB403 GCG + katG203 ACC	gyrB403 TCG + katG203 ACT
*M. africanum*	ethA124 GAC + nt1673338 A or	ethA124 GGC + nt1673338 C
	inhA78 GCG + atpE69 GCT	inhA78 GTG + atpE69 GCG
*M. pinnipedii*	inhA107 TCG + nt1473094 C	inhA107 CCG + nt1473094 G
*M. microti*	gyrB144 TAT + nt1473079 A	gyrB144 TAC + nt1473079 C
*M. caprae*	gyrB171 GTA + gyrB356 GCG	**gyrB171 GTA** + gyrB356 GCT
*M. bovis* bacillus Calmette-Guérin	pncA57 GAC + furA43 GTC	pncA57 CAC + furA43 GCC
*M. bovis*	pncA57 GAC + furA43 GCC	pncA57 CAC + **furA43 GCC**
Lineage		
1 (Indo-Oceanic)	gidB110 GTT	gidB110 GTG
2 (Beijing)	gidB92 GAC	gidB92 GAA
3 (Central-Asian)	nt2726105 A	nt2726105 G
4 (Euro-American)	katG463 CGG	katG463 CTG
5 (West African 1)	ethA124 GAC	ethA124 GGC
6 (West African 2)	inhA78 GCG	inhA78 GTG

*The codons or individual nucleotide positions were based on the sequence of the reference genome H37Rv. None of the complete marker set are present in strain 20-2359; 2 partial matches are in bold. Three primate (SRR1792164, SRR1792165, SRR7617662) and 2 elephant (DRR120408, DRR120409) isolates shared the same genotypes with 20-2359 for these markers.

SNP-based phylogeny with 100% bootstrap support using *M. tuberculosis* H37Rv as a reference placed 20-2359 close to isolates from 3 primates (NCBI SRA nos. SRR1792164, SRR1792165, SRR7617662) and 2 elephants kept in captivity in Japan (NCBI SRA nos. DRR120408, DRR120409) (Figure). These 6 sequences form a distinct group that branches halfway between the *M. bovis* La1.1 and *M. caprae* La2 clades. SNP distances between members of the same clade (*M. caprae*, *M. bovis*, or La1.1) all differed by <802 SNPs, whereas SNP difference across clades averaged 1,369 (range: 985–1,374 SNPs) (Figure). Within the 20-2359 cluster, the maximum SNP distance between any 2 isolates was 776. The number of SNPs between the 20-2359 cluster and any *M. caprae,* La1.1, or *M. bovis* bacillus Calmette-Guérin strain averaged 1,161; the minimum was *M. caprae* SRR13888754 with 1,047.

**Figure:**
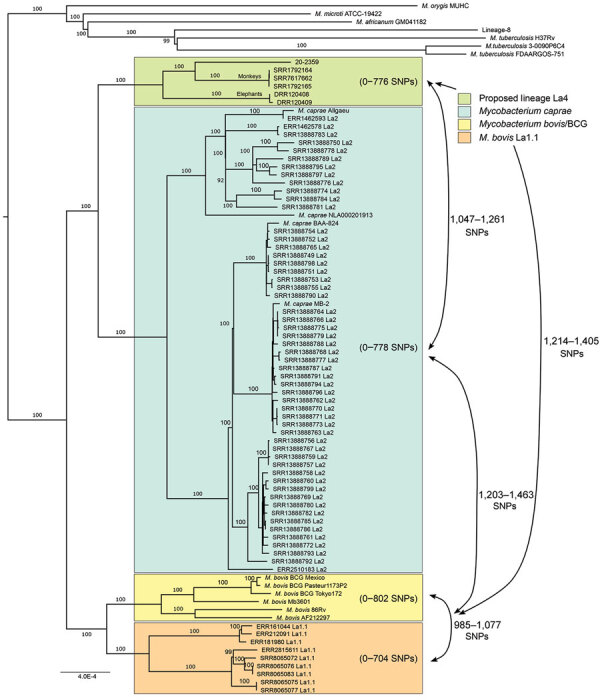
Phylogenetic SNP tree of strain 20-2359 and diverse group of representative *Mycobacterium caprae*, *M. bovis*, and other species and strains gathered from publicly available databases. Phylogenetic tree was calculated from the SNP alignment using IQ-TREE 1.6.12, with automatic best model selection (TVM+F+ASC+R2 model), and with 1,000 bootstrap support calculations (*18*). We used 14,688 variable genomic sites for this analysis. BCG, bacillus Calmette-Guérin; SNP, single-nucleotide polymorphism.

## Discussion

We identified *Mycobacterium* strains through WGS based on a combination of results from genomic database comparisons, spoligotype analysis, and detection of lineage-specific markers; each method has unique limitations. Although results generated by these methods usually agree, rare or unknown genotypes, not represented or improperly labeled in databases, can result in discordance and require a more in-depth analysis for final identification. When we first received sample 20-2359, initial presentation and culture testing did not indicate an atypical bacterium. However, when we first screened RD to confirm the strain identity, we noticed weaker amplification of some targets and the absence of RD9, indicating that the strain might belong to a less-common species or lineage within MTBC. Our attempts at identifying the strain through WGS analysis using results from Kraken, in-house lineage-specific markers, and in-silico spoligotyping all indicated it was somewhat related to *M. bovis* or *M. caprae*, but not which species or lineage.

SNP-based phylogenetic analyses using our local database, which contains >4000 clinical and nonclinical strains (data not shown), placed 20-2359 in a distinct lineage, a sister to *M. caprae* and more distantly related to *M. bovis*. A more focused phylogenetic analysis of publicly available sequences of animal-associated *M. caprae*, *M. bovis*, and other *Mycobacterium* spp. revealed that 20-2359 formed a well-supported cluster with 3 primate and 2 elephant isolates, distinct from *M. caprae*, *M. bovis*, and La1.1 (Figure). La1.1 is a newly classified animal-associated sublineage of *M. bovis* that is pyrazinamide susceptible, having branched off before acquisition of the pncA H57D mutation found in nearly all *M. bovis* strains worldwide, as described elsewhere (*14*). By comparing SNP counts between the 20-2359 cluster and the other isolates (Figure), we confirmed the distinctive nature of this cluster. The range of SNP distances (1,047–1,405) between isolates forming the 20-2359 cluster (proposed lineage La4) and isolates from other clades was lower than that between *M. caprae* and *M. bovis* and La1.1 isolates (1,203–1,463) but higher than that between *M. bovis* and La1.1 subclade isolates (985–1,077). Phylogenetic placement of proposed lineage La4 strains, along with the SNP distances to other clades, strongly suggests that isolates from this cluster belong to a new MTBC lineage associated with mammals from eastern and southeastern Asia.

We found the arrangement of RD4 in our clinical strain, 20-2359, unique from closely related primate and elephant isolates, which had complete RD4 gene clustering identical to *M. caprae* variant Allgaeu and other strains. Similarly, 20-2359 shared a spoligotype only with the 3 strains from primates, whereas the 2 strains from elephants had a spoligotype sequence with 1 extra spacer at spacer 2, identical to a spoligotype from the *M. caprae* clade. These differences within members of the 20-2359 cluster might reflect geographic diversity or differences in animal reservoirs. Limited information was available regarding the 3 MTBC samples from primates except that they were isolated in China. NCBI SRA nos. DRR120408 and DRR120409 samples were isolated at 2 time points from an elephant originally from the island of Borneo living in captivity in a zoo in Japan (*20*).

We could not establish the exact origin of clinical isolate 20-2359 based on available patient information; however, the patient grew up in Bangladesh and had potentially contracted TB through consuming raw milk. Results from a 2016 study reporting detection of *M. caprae* in 44 swamp buffalos from 4 farms in Thailand suggest that this strain type might have been encountered in the past (*21*). However, in that report, identification was based solely on spoligotype, which we have shown is conserved between some *M. caprae* strains and the new proposed lineage. The geographic location in that report is particularly intriguing given it is not typical for *M. caprae*. Although not possible to confirm with the available data, one possibility is that the swamp buffalo were infected not with *M. caprae* but with this newly described sister lineage. Given the distinct phylogenetic placement of this cluster, relatively long SNP distances to all *M. bovis*, La1.1, and *M. caprae* isolates in our dataset, and the case-patient’s geographic origin, which was atypical for the presence of *M. caprae*, we propose cluster 20-2359 belongs to a new MTBC lineage, La4, based on new nomenclature for animal-adapted MTBC lineages (*14*).

Appendix Additional information about novel lineage of *Mycobacterium* species.
